# Societal Attitudes Towards Autism (SATA): Validation of the Greek Version in the General Population

**DOI:** 10.1007/s10803-022-05842-2

**Published:** 2023-01-10

**Authors:** Vasiliki Zarokanellou, Alexandros Gryparis, Paraskevi Papatheodorou, Giorgos Tatsis, Dionysios Tafiadis, Angelos Papadopoulos, Louiza Voniati, Vassiliki Siafaka

**Affiliations:** 1 Department of Speech and Language Therapy, School of Health Sciences, University of Ioannina, 4th Km National Road Ioannina-Athens, 45500 Ioannina, Greece; 2 Karamandaneio Prefecture Children Hospital of Patras, Patras, Greece; 3 Department of Speech & Language Therapy, Faculty of Sciences, European University, Nicosia, Cyprus

**Keywords:** Societal attitudes, Knowledge, Autism Spectrum Disorders, Validation, General population

## Abstract

This study examined the validity of the Greek version of the Societal Attitudes Towards Autism (SATA) scale in a Greek community sample (*n* = 633) and explored how the demographic variables of the sample modulate knowledge and attitudes regarding people with Autism Spectrum Disorder (ASD). The principal component analysis confirmed the three-dimension model and explained 40.5% of the variance. All Cronbach’s alpha values obtained were over 0.70. SATA’s subscales were significantly and positively correlated, indicating good internal reliability. Participants presented moderate knowledge about ASD and mediocre positive attitudes towards people with ASD. Gender, age, and educational level significantly affected SATA total scores. Overall, this Greek version of SATA showed acceptable psychometric properties, indicating that can be a reliable scale for use.

## Introduction

Autism Spectrum Disorder (ASD) is a complex neurodevelopmental disorder that is characterized by persistent deficits in social communication and social interaction across multiple contexts, as well as restricted and repetitive patterns of behaviour such as insistence on sameness, inflexible adherence to routines, motor stereotypies, echolalia, etc. (DSM-5, 2013). ASD is a lifelong condition with its symptoms be presented in the early developmental period (Volkmar & Jackson, 2020). In the last 30 years, epidemiological studies documented a dramatic rise in the prevalence of ASD with its global prevalence increasing from 62 to 10,000 (one in 162) in 2012 to 65 per 10,000 (one in 154) in 2021 (Zeidan et al., 2022). In Greece, the only prevalence study was conducted by the Centers for Educational and Counseling Support in 2019, examining 10–11 years old children eligible to receive special educational support in school settings, reported that the nationwide prevalence of ASD was 115 per 10,000 (one in 87) (Thomaidis et al., 2020). According to researchers, approximately, 33–35% of children with ASD are also classified as having an intellectual disability (IQ < 70) implying that most of these children attend regular elementary schools (Maenner et al. 2021; Zeidan et al., 2022). The above suggests that more people now and in the future will interact with individuals with ASD in educational settings, in everyday activities and in the workplace; thus, it is important to learn more about the public’s orientation towards people with ASD and understand better the public’s attitudes towards this specific population.

### Knowledge and Attitudes of the General Population Regarding People with ASD and Their Family Members

People with ASD face social stigma, prejudice, and discrimination to the same extent as people with physical or mental disabilities, even though ASD is not always a visible disability (Dachez et al., 2015; Obeid et al., 2015). Individuals with ASD usually present maladaptive social behaviours and sensory deficits even as adults and frequently perceive public criticism emerging from misconceptions about their behaviour (Milačić-Vidojevic et al., 2014). Furthermore, family members of people with ASD often experience courtesy stigma via their connection with the person with ASD, which frequently leads to the development of affiliate stigma, resulting in social withdrawal or concealment, and poor family quality of life (Milačić-Vidojevic et al., 2014; Mitter et al., 2019; Papadopoulos et al., 2022). In Greece, there is very limited research regarding the attitudes towards people with ASD. Two recent qualitative studies on primary caregivers of preschool and school-age children with ASD reported that most parents of children with ASD had experienced prejudice or negative stereotypes (Veroni, 2019; Papadopoulos, 2021). Specifically, parents reported that their child had been stigmatized as ‘disobedient’ and in high percentage (18.9%) it had experienced bullying (Veroni, 2019). Furthermore, the studies reveal that mothers of preschool children with ASD or children who had been newly diagnosed with ASD face moderate levels of stigma, which has a negative and significant effect on the social life of their family (Papadopoulos, 2021; Papadopoulos et al., 2022).

Previous evidence supports that knowledge about ASD and social contact with people with ASD influence positive attitudes toward people with ASD and their families (Chu et al., 2021; Dachez et al., 2015; Gemegah et al., 2021; Kuzminski et al., 2019). Several researchers have investigated the knowledge about ASD and the attitudes of unaffected people towards people with ASD in community samples from different cultures (Cage et al., 2019; Chu et al., 2021; Dachez et al., 2015; Dickter et al., 2020; Durand-Zaleski et al., 2012; Gemegah et al., 2021; Jensen et al., 2016; Jones et al., 2021; Kuzminski et al., 2019; Surmen et al., 2015; Wang et al., 2012). Studies from developed countries with individualist communities such as Great Britain, the USA, and Australia reveal higher levels of knowledge about ASD and lower levels of stigma (Cage et al., 2019; Jones et al., 2021; Kuzminski et al., 2019; Obeid et al., 2015), compared to studies from developing and underdeveloped countries with collectivist communities such as Malaysia, Turkey, and China, which reveal moderate or low levels of knowledge and higher levels of stigma (Chu et al., 2021; Surmen et al., 2015; Wang et al., 2012). Most research has proposed that participants held basic knowledge about the disorder but had several misconceptions about the difficulties people with ASD face or the support they need (Durand-Zaleski et al. 2012; Jensen et al. 2016; Jones et al., 2021; Wang et al., 2012). Additionally, the majority of research suggests that the higher education level of participants (Chu et al., 2021; Jones et al., 2021), the specific knowledge about ASD, and the social contact with people with ASD (Chu et al., 2021; Dachez et al., 2015; Gemegah et al., 2021; Jensen et al., 2016; Kuzminski et al., 2019), as well as the female gender (Cage et al., 2019; Jensen et al., 2016; Kuzminski et al., 2019; Obeid et al., 2015; Wang et al., 2012) are generally associated with more positive attitudes towards individuals with ASD. However, the effect of gender is not consistent in all studies (Jones et al., 2021; Matthews et al., 2015).

Furthermore, it is well known that socioeconomic disparities contribute to inequalities in access to ASD resources within and between countries (Grinker et al., 2011; Papadopoulos, 2016) and inadequate resources may contribute to stigma. The United States has higher levels of resources in general and ASD resources in particular than Greece (through the Greek National Insurance Service). However, most children with ASD in Greece also attend costly interventions by specialists, either at home or at outpatient centers (Stampoltzis et al., 2012).

In addition, the medical model has been widely adopted in Greece for a coherent understanding of the ASD and implementation of intervention programs for individuals with ASD (Kossyvaki, 2021). According to the medical model, the deficits lie within the individuals with ASD who have to be ‘treated’ while the school environment and the social setting are set and, therefore, difficult to change in order to cover their needs (Rieser & Mason, 1990).

Undoubtedly, in Greece, there is a need for higher understanding and acceptance of Autism Spectrum Disorders. The fact that parents often withhold an ASD diagnosis due to fear of stigma highlights the need for targeted interventions, based on evidence, in the general population to improve knowledge about ASD and change thoughts and feelings towards people with ASD (Kossyvaki, 2021).

### Measurements Assessing Attitudes Towards People with ASD

There are very few measurements to assess attitudes toward individuals with ASD, namely the Societal Attitudes Toward Autism (SATA) Scale (Flood et al. 2013) and the Multidimensional Attitude Scale Toward Persons with Disabilities (MAS) (Flinder et al., 2007). The Multidimensional Attitude Scale Toward Persons with Disabilities (MAS) (Flinder et al., 2007) is a short scale, which assesses attitudes towards people with disabilities and has been adapted for people with ASD in French (Dachez et al., 2015), American English (Matthews et al., 2015) and Chinese (Lu et al., 2020). SATA is the first instrument that was validated to assess knowledge and social attitudes towards ASD and it consists of three subscales (Societal Attitudes, Knowledge, and Personal Distance). Even though SATA is the only scale merely validated to evaluate explicit attitudes towards people with ASD, it has not been translated and adapted into any other language than Malay (Low et al., 2018). The original English version of the scale shows acceptable reliability levels with an overall Cronbach alpha of 0.77 for the total scale. However, the three subscales of the instrument were inconsistently related to one another (Flood et al., 2013). The convergent validity of the scale was explored through correlations with the Attitudes towards Disabled Persons (ATDP), the Preference for Persons with Autism Statement, the Disability Attitudes Implicit Association Test (DA-IAT), and the Behavioral Intention. All the subscales of the SATA showed positive correlations with the DA-IAT indicating that SATA, which is an explicit scale estimating the products of a conscious process, is associated with implicit attitudes (evaluations that are outside of conscious awareness as a result of past experiences), but it is also susceptible to social desirability. Furthermore, the Societal Attitudes and the Knowledge subscales of the original scale both showed medium positive associations with the attitudes towards people with disability as measured by the ATDP and autism diagnosis as measured by the Preference for Persons with Autism Statement (Flood et al., 2013). Finally, the validity study of the test revealed that experts had a higher level of acceptance and more refined knowledge of ASD disorders than undergraduate students. However, the Knowledge and Personal distance subscales yielded inconsistent reliability and validity outcomes. Until today, no other study has examined the psychometric properties of SATA in any other language than English, thus, more evidence is needed about its validity and reliability.

Taking the above into consideration, the purpose of the present study is threefold. Firstly, to translate and adapt the Societal Attitudes towards Autism (SATA) scale in Greek and to validate it, since it is the only standardized scale, which assesses knowledge and attitudes regarding this specific population. Secondly, to evaluate the knowledge and attitudes of a Greek community sample regarding people with ASD. Finally, to investigate the differences in SATA scores for demographic variables (such as gender, age, educational level, etc.) in a Greek community sample. Using a validated instrument in Greek, the findings of the current study will contribute to the planning of interventions to improve the knowledge and awareness about ASD in targeted groups of the Greek general population in order to prevent stigma experiences and improve the quality of life of people with ASD and their families.

## Methods

### Participants

Participants were 633 individuals, who agreed to participate voluntarily in the current study. The inclusion criteria included people older than 18 years of age, unaffected of ASD, without physical, mental, intellectual or sensory impairments and permanent residents of Greece. The sample consisted of 107 men (16.9%), and 526 women (83.1%) from various social and educational backgrounds. The age range of participants were: ≤30 years (45.0%), 31–50 years (44.2%), > 50 years (10.8%). Most of the participants had completed at least university studies (71.7%) and lived in urban or semi-urban areas of Greece (87.8%). Additionally, 37.8% of the sample were parents and 62.2% did not have a child. Finally, 23.4% of the participants had a relative or a friend with ASD. Analytically, the sample’s characteristics are presented in Table 1.

**Table 1:** Sample’s sociodemographic characteristics

	Number of subjects	%
Gender		
Male	107	16.9
Female	526	83.1
Age group		
≤ 30 years old	285	45.0
31–50 years old	280	44.2
> 50 years old	68	10.8
Educational level		
Elementary school	13	2.1
Junior High / High school	166	26.2
Bachelor’s or higher	454	71.7
Residency		
Urban areas	556	87.8
Rural areas	77	12.2
Relative/friend with ASD		
Yes	148	23.4
No	485	76.6
Monthly income		
< 400 €	48	7.6
400–800 €	112	17.7
800-1,500 €	277	43.8
1,500-2,000 €	101	16.0
> 2,000 €	95	15.0
Occupation		
Teacher (Elementary-High School level)	123	19.4
Health or Mental Health professional	159	25.1
Civil servant	36	5.7
Private sector employee / Freelancer	121	19.1
Farmer	16	2.5
Student	145	22.9
Unemployed	33	5.2
Children		
Yes	239	37.8
No	394	62.2
Total	633	100.0

### Instrument

#### Description of SATA

The original scale is composed of three factors and contains 26 items, in which the responses are given using a 4-point Likert scale (1 = *strongly disagree* to 4 = *strongly agree*) (Flood et al., 2013). The first factor assesses Social Attitudes towards ASD and includes 16 items (range score 16–64). The second factor evaluates Knowledge about the disorder (5 items, range score 5–20) and the third factor examines Personal Distance that refers to the extent to which participants are willing to interact with individuals with ASD across situations (5 items, range score 5–20). Lower scores indicate better knowledge and positive societal attitudes regarding individuals with ASD. Regarding Personal Distance, higher scores correspond to more willingness to engage with people with ASD.

#### Translation and Adaptation of SATA

The translation and adaptation of SATA were performed according to the Minimal Translation Criteria (MOT, 1997) as follows: (a) The instrument was translated into Greek by two independent proficient bilingual speakers, who discussed and agreed on a reconciled Greek version of the test. (b) The Greek version of the instrument was back-translated into English by an independent professional translator. (c) The back-translation was reviewed, and cognitive debriefing procedures were performed by the members of the research team, who are proficient bilingual speakers of English and Greek, establishing a pre-final version of the questionnaire. Finally, the comprehensibility of the questionnaire was checked by asking ten non-professionals to review the items of the questionnaire and minor modifications were made, leading to the final version of the questionnaire.

### Procedure

Before the beginning of the study, we have adhered to the guidelines of the Research and Ethics Committee of the University of Ioannina, and we have obtained all required approvals for the study. Furthermore, we have obtained authorization for the translation and the research use of the scale from the authors of the questionnaire. Participants were informed for the present study via social media and agreed to participate voluntarily in the study. Data were collected through an online survey using snowball sampling techniques during one month period time. These included the distribution of the questionnaire via Google form to several social platforms like Facebook and Viber, as well as its distribution to personal contacts via email and Viber. The snowball sampling method is purely based on referrals and involves a primary source nominating other potential data sources that will be able to participate in a study. Participants with missing data on the SATA questionnaire, who presented a disability or were younger than 18 years old were excluded from all the analysis.

### Statistical Analysis

Since SATA used a four-point Likert scale, responses of participants were coded such that a high score indicates high acceptance or high knowledge (e.g., 4 points for the answer “Strongly Agree”). For the statistical analysis of the Greek version of the scale, we used reverse coding for all items except for items 2 “People with autism should have the opportunity to go to college”, 9 “I would be comfortable sitting next to a person with autism in the same class”, and 13 “Children with autism should be fully integrated into mainstream classes” of the Societal Attitudes subscale, and for all items of the Personal Distance subscale, following the suggestion of the original version (Flood et al., 2013). Categorical variables are presented as absolute and relative (%) frequencies. Quantitative variables are presented as mean ± SD. In terms of graphical representation, quantitative variables are presented with boxplots, when needed. To examine the internal consistency and reliability of the instrument Cronbach’s alpha coefficient was used. The analysis was performed for each of the subscales, and overall. Furthermore, we investigated the value that Cronbach’s alpha would be if each particular item was subsequently deleted from the scale. In the case that the deletion of an item resulted in an increased Cronbach’s alpha, that would suggest that possibly the specific item should be removed from the list of items, since its exclusion results in a considerably improved internal validity. To investigate the construct validity of the Greek version of the SATA scale, a principal components factor analysis was implemented, using a varimax rotation and a Kaiser normalization. Regarding testing the assumptions of the principal components analysis, the Kaiser-Meyer-Olking (KMO) statistic should be greater than 0.600 and the Bartlett’s test should be significant (i.e., p-value < 0.05). To compare SATA total scores between two or more groups, Mann-Whitney test and Kruskal-Wallis test were employed, respectively. Spearman’s correlation coefficient was implemented to evaluate the linear relationship between different SATA subscales. A two-tailed p-value < 0.05 was considered statistically significant. Statistical analysis was implemented using ΙΒΜ SPSS v. 28 (IBM Corp. Released 2021. IBM SPSS Statistics for Windows, Version 28.0. Armonk, NY: IBM Corp.).

## Results

**Fig. 1:**
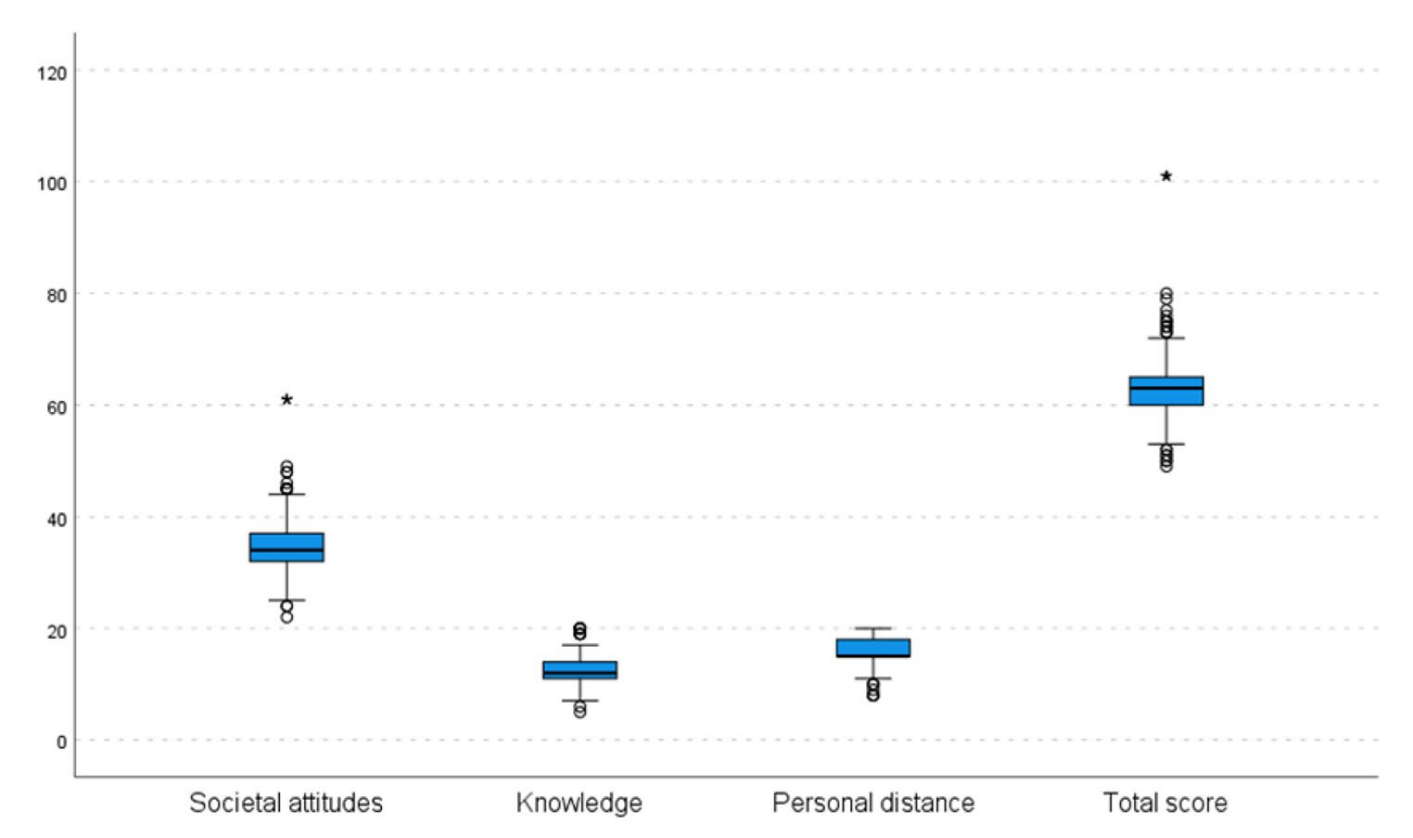
Boxplot of the SATA questionnaire subscales and total scores Circles Indicate Outliers; Asterisks Indicate Extreme Outliers

The statistical analysis of the data revealed moderate levels of Knowledge and mediocre levels of positive Societal Attitudes toward ASD, with mean scores of knowledge being 12.3 ± 2.0 and of attitudes being 34.5 ± 4.4, but rather high levels of willingness to engage in regular and close relationships with people with ASD, with the mean score on the Personal Distance subscale being 15.9 ± 2.4 (Table 2; Fig. 1).

**Table 2:** Mean scores and Standard deviations on SATA

Subscale	N	Mean	Median	SD	Min	Max
Societal Attitudes	633	34.5	34.0	4.4	22.0	61.0
Knowledge	633	12.3	12.0	2.0	5.0	20.0
Personal Distance	633	15.9	15.0	2.4	8.0	20.0
Total Score SATA	633	62.8	63.0	5.1	49.0	101.0

### Internal Consistency and Reliability

An overall standardized Cronbach’s alpha of 0.872 was obtained, indicating good internal reliability (Nunnally, 1978). Cronbach’s alpha values depicting the level of internal consistency for each of the subscales were as follows: Societal Attitudes (*a* = 0.812), Personal Distance (*a* = 0.765) and Knowledge (*a* = 0.843). Furthermore, the subscales were consistently related to one another (Table 3) with a statistically significant and positive Spearman’s correlation coefficient.

**Table 3:** Spearman’s correlation coefficient between the subscales of SATA

Subscale	Societal Attitudes	Knowledge	Personal Distance
**Societal Attitudes**	1.000	0.447*	0.617 *
**Knowledge**		1.000	0.334*
**Personal Distance**			1.000

* P-value < 0.001

Additionally, we investigated the value that Cronbach’s alpha would be if every particular item was deleted from the scale. For the majority of the items, the removal of each item would result in a lower Cronbach’s alpha for that specific subscale, with the following exceptions:

- For the subscale ‘Societal attitudes’ the item “Children with autism should be fully integrated into mainstream classes.”
- For the subscale ‘Personal Distance’ the item “People with autism are capable of living normal lives (i.e., with a job, house, family, etc).”
- For the SATA total score the items “People with autism are incapable of forming relationships and expressing affection.” and “People with autism need assistance communicating with others.”

For these items, the marginal increased Cronbach’s alpha values after their removal lead us to not consider removing these items.

### Construct Validity

The Kaiser-Meyer-Olking statistic was equal to 0.882 and the Bartlett’s test p-value was lower than 0.001, confirming the model’s assumptions. Furthermore, principal components analysis revealed that for the model of three principal components which was suggested by Flood et al., (2013) the total % of variance explained is approximately 40.5%. The communalities of the individual items ranged from 15 to 74% (see Appendix Table A1).

The analysis of the current study showed that 12 items, specifically items 1, 2, 3, 4, 5, 7, 8, 10, 11, 12, and 14 from the original Societal Attitudes subscale and item 3 from the original Personal Distance subscale loaded on the first factor. Similarly, on the second factor loaded 5 items, which are items 1, 2, 3, and 4 from the original Personal Distance subscale, as well as item 9 from the original Societal Attitudes subscale. Finally, on the third factor loaded 9 items. These items are: items 6, 13, 15 and 16 from the original Societal Attitudes subscale, items 1, 2, 4, and 5 from the original Knowledge subscale, and item 5 from the original Personal Distance subscale” (see Appendix Table A2).

### Differences in SATA Scores for Demographic Variables

The statistical analysis revealed that Societal Attitudes towards people with ASD and the overall SATA score differ by age group (*p* < 0.001 and *p* = 0.006, respectively), gender (*p* = 0.035 and *p* = 0.048, respectively), and educational level (*p* < 0.001 and *p* = 0.004, respectively), as shown in Table 4. On the contrary, area of residency, previous social contact with people with ASD, and familial monthly income did not significantly impact the overall score of the SATA scale (Table 4). Analytically, as presented in Table 4, women showed higher levels of knowledge and significantly more positive attitudes toward people with ASD, scoring lower on the SATA Total score (62.6 ± 5.1) than men (63.5 ± 4.7) (*p* = 0.048). Age was significantly associated with the score on the SATA scale with younger people demonstrating higher levels of knowledge and more positive attitudes towards people with ASD than older ones (> 50 years) *(p* = 0.006). Specifically, participants aged 30–50 years old had lower Total score presenting higher levels of knowledge and openness towards individuals with ASD, while respondents younger than 30 years, scored higher on the Personal Distance subscale, demonstrating more willingness to engage on a regular basis with people with ASD than the two other age groups (*p* < 0.001) (Table 4).

**Table 4:** Descriptive statistics of SATA subscales and total scores, and associations with sociodemographic characteristics

Sociodemographic characteristics			SATA subscales				SATA Total Scores	
	Societal Attitudes		Knowledge		Personal Distance			
	Mean (SD)	P-value	Mean (SD)	P-value	Mean (SD)	P-value	Mean (SD)	P-value
Gender		0.035*		0.267		0.263		0.048*
Male	35.3 (4.5)		12.5 (2.0)		15.6 (2.6)		63.5 (4.7)	
Female	34.3 (4.4)		12.3 (2.0)		16.0 (2.4)		62.6 (5.1)	
Age group		< 0.001 *		< 0.001 *		< 0.001 *		0.006 *
≤ 30 years old	33.8 (4.7)		12.4 (2.0)		16.5 (2.5)		62.7 (5.7)	
31–50 years old	34.6 (3.8)		12.1 (1.9)		15.7 (2.2)		62.4 (4.2)	
> 50 years old	37.3 (4.3)		13.1 (2.3)		14.3 (2.2)		64.6 (4.9)	
Educational level		< 0.001 *		< 0.001 *		0.001 *		< 0.004 *
Elementary school	40.9 (5.5)		14.2 (1.8)		13.3 (2.2)		68.4 (5.9)	
Junior High / High school	34.1 (4.2)		12.6 (2)		16 (2.6)		62.7 (4.4)	
Bachelor’s or higher	34.5 (4.3)		12.2 (2)		16 (2.3)		62.6 (5.2)	
Residency		0.156		0.028 *		0.633*		0.190
Urban areas	34.3 (3.8)		12.2 (1.9)		16.0 (2.3)		62.5 (4.1)	
Rural areas	36.2 (7.3)		13.0 (2.6)		15.7 (3)		65.0 (9.2)	
Relative/friend with ASD		0.264		0.063		0.056		0.749
Yes	34.0 (3.8)		12.0 (1.8)		16.3 (2.4)		62.3 (4)	
No	34.7 (4.5)		12.4 (2.1)		15.8 (2.4)		62.9 (5.3)	
Monthly income		0.196		0.182		0.581		0.179
< 400 €	35.6 (4.7)		12.8 (1.7)		15.9 (2.7)		64.4 (5.0)	
400–800 €	34.5 (4.2)		12.2 (2.1)		15.8 (2.5)		62.5 (4.4)	
800-1,500 €	34.5 (3.8)		12.2 (1.8)		15.8 (2.4)		62.5 (4.1)	
1,500-2,000 €	33.7 (3.6)		12.4 (2.2)		16.1 (2.3)		62.2 (4.1)	
> 2,000 €	34.9 (6.3)		12.5 (2.3)		16.3 (2.4)		63.7 (8.1)	
Occupation		0.008 *		0.559*		0.002 *		0.203 *
Teacher (Elementary- High School level)	34.5 (3.9)		12.3 (1.9)		15.9 (2.3)		62.7 (4.4)	
Health or Mental Health professional	34.8 (3.4)		12.1 (2.1)		16.0 (2.3)		62.9 (4.1)	
Civil servant	34.9 (4.0)		12.3 (1.4)		15.5 (2.3)		62.7 (3.2)	
Private sector employee / Freelancer	35.0 (6.1)		12.3 (2.3)		15.5 (2.4)		62.8 (7.7)	
Farmer	37.1 (4.6)		12.9 (2.2)		14.4 (1.5)		64.4 (5.7)	
Student	33.4 (3.9)		12.5 (2.0)		16.6 (2.5)		62.4 (4.2)	
Unemployed	34.7 (4.2)		12.5 (2.2)		16.1 (2.4)		63.2 (4.5)	
Children		< 0.001 *		0.698		< 0.001 *		0.830
Yes	35.2 (4.2)		12.4 (2)		15.2 (2.2)		62.8 (4.7)	
No	34.1 (4.4)		12.3 (2)		16.4 (2.4)		62.8 (5.3)	

SD: Standard Deviation, * Statistically significant results

Moreover, the respondents with higher educational levels (Bachelor’s degree or higher) showed increased levels of knowledge about ASD (*p* < 0.001), more positive attitudes towards people with ASD (*p* < 0.001), and more openness to contact socially with them than participants with lower academic levels (*p* = 0.001). Surprisingly, the results did not reveal a significant association of previous experience with people with ASD on the mean scores in any of the three subscales of the SATA scale or overall, on the mean Total score (*p* = 0.749).

Participants who lived in urban areas exhibited significantly higher levels of knowledge (12.2 ± 1.9) about people with ASD than participants who lived in rural areas (13.0 ± 2.6) (*p* = 0.028). Additionally, they also scored higher on the subscale of Personal Distance, implying that they were more ready to contact socially with people with ASD than respondents from rural areas, but overall, the area of residency did not associate with the overall score on the SATA scale (*p* = 0.190) (Table 4). Moreover, the farmers and the unemployed, had the higher Total score on the SATA scale, displaying the lower levels of knowledge and the more negative attitudes (*p* = 0.008) towards people with ASD.

Finally, the respondents who were parents had significantly more positive attitudes towards people with ASD than the nulliparous (*p* < 0.001) but were significantly more reluctant to communicate regularly and closely with people with ASD than those without children (*p* < 0.001).

## Discussion

The current study attempted to translate and adapt the SATA scale (Flood et al., 2013) into Greek, as well as to validate the Greek version of the questionnaire, which to the best of the authors’ knowledge comprises the first validity study of the instrument in another language than English. Furthermore, the study aimed to investigate the knowledge and attitudes regarding people with ASD in a Greek community sample and to explore how the factors of age, gender, educational level, income, occupation, previous social contact with people with ASD, and parenthood moderated scores on the SATA scales.

As it concerns, the Greek version of SATA presents acceptable levels of internal consistency with Cronbach’s alpha values for all subscales yielding over 0.75, in contrast to the original scale (Flood et al., 2013) in which the subscale of Knowledge yielded an unsatisfactory alpha coefficient lower than 0.50. Moreover, the Greek adaptation of the instrument shows acceptable reliability levels since the three subscales of the instrument are statistically significantly and positively correlated with each other showing better psychometric properties than the SATA original scale (Flood et al., 2013). Analytically, the subscale of Societal Attitudes shows moderate and positive correlations with the subscales of Knowledge and Personal Distance, meaning that better knowledge of ASD is associated with more positive attitudes towards people with ASD, and positive attitudes towards people with ASD are significantly related to openness to engage in social relationships with them. Contrary to the original validation study (Flood et al.,2013), in which the subscales of Personal Distance and Knowledge were not significantly correlated, in the current study the two subscales present a significant weak association, implying that higher levels of knowledge of ASD, may be associated with more willingness to interact with people with ASD. Furthermore, the removal of the majority of items of the scale returns a lower Cronbach’s alpha value with the exception of two items for the overall scale, which their removal returns a marginal increase in the Cronbach’s alpha values, making us consider that there is no real need to be removed. The Greek version of the SATA scale also presents acceptable construct validity since the Kaiser-Meyer-Olking statistic was equal to 0.882 and the Bartlett’s test p-value was lower than 0.001, indicating that our correlation matrix is not an identity matrix, as was supposed according to the original model (Flood et al., 2013). The exploratory factor analysis explained approximately 40.5% of the variance for the three-structure model suggested by Flood et al., (2013), a significantly higher percentage of variance in comparison to the original scale (Flood et al., 2013), with the communalities of items ranging from 15 to 74%. Furthermore, based on the factor analysis most of the items (11 out of 16) that correspond to the Societal Attitudes Scale loaded on the 1st factor which seems to depict Societal Attitudes attributes. The 2nd factor included all items from the Personal Distance subscale; hence, the 2nd factor depicts personal distance attributes. The 3rd factor included all Knowledge subscale items, along with some items mostly from the Societal Attitudes subscale. In this last factor, there seems to be a mixture between Knowledge and Societal Attitudes subscales. The above reveals that the subscale of Knowledge needs additional research in order to become a reliable measure for the purpose of measuring knowledge for ASD, a finding which is consistent with the results from the original standardization of the tool (Flood et al., 2013).

In general, the study participants showed mediocre levels of knowledge and moderate positive attitudes regarding people with ASD, a result which comes in accordance with the findings from other similar studies in developing and developed countries (Chu et al., 2021; Jensen et al., 2016; Wang et al., 2012). Relevant research (Chu et al., 2021) suggests that misconceptions about the difficulties that people with ASD experience, result in poor levels of community support, while interventions targeting knowledge about ASD may improve attitudes and increase acceptance, resulting in a better quality of life for people with ASD and their families. Moreover, Grinker et al., (2011) conclude that high quality service provisions are in general more likely to exist in countries or communities with a high level of understanding and awareness towards autism, in comparison with social contexts where awareness is low.

Results also validated that gender would significantly affect scores on the Greek SATA scale. Specifically, women hold significantly more positive attitudes toward people with ASD than men, scoring significantly lower on the Societal Attitudes scale and overall, on the Greek SATA scale, a result which replicates findings from previous studies (Cage et al., 2019; Jensen et al., 2016; Kuzminski et al., 2019). It is argued that the female gender significantly and positively affects attitudes toward people with ASD (Cage et al., 2019; Jensen et al., 2016; Kuzminski et al., 2019; Obeid et al., 2015; Wang et al., 2012), but this finding is not consistent in all studies (Jones et al., 2021; Matthews et al., 2015). Women’s positive attitudes may be attributed to their level of empathy, as most studies on empathy indicate that women show higher levels of empathy than men taking into consideration contextual factors and stereotypical beliefs (Löffler & Greitemeyer, 2021).

Furthermore, younger participants had significantly higher scores in all the subscales of the Greek SATA scales, as well as significantly lower overall scores. This confirms previous findings from comparable studies in different countries (Jones et al., 2021; Wang et al., 2012). Generally, unaffected individuals were more open to involving in distant social relationships than in intimate relationships with people with ASD and rejected the idea of having a romantic affair or a boss with ASD (Jensen et al., 2016; Obeid et al., 2015).

In addition, the statistical analysis confirmed that the higher educational level of participants is significantly correlated with higher levels of knowledge about ASD and more openness towards people with ASD, reinforcing similar findings from a body of previous research (Chu et al., 2021; Jones et al., 2021). Recent evidence concluded that older unaffected individuals with lower education levels were more likely to believe that people with ASD are violent and should attend a special school (Jones et al., 2021), while others did not find such an association (Kuzminski et al., 2019).

On the contrary, the results of the present study did not reveal a significant effect of previous experience / social contact with people with ASD on SATA scores. The above finding opposes relevant research (Chu et al., 2021; Dachez et al., 2015; Gemegah et al., 2021; Jensen et al., 2016; Kuzminski et al., 2019), which concluded that specific knowledge about ASD and social contact with people with ASD significantly predict more positive attitudes towards individuals with ASD (Chu et al., 2021; Dachez et al., 2007; Gemegah et al., 2021; Jensen et al., 2016; Kuzminski et al., 2019). However, for the interpretation of this finding, it is of the note that SATA (Flood et al., 2013) is an explicit scale estimating the products of a conscious process. It has not been designed to access implicit attitudes which include evaluations that are outside of conscious awareness as a result of past experience which continues to influence behavior. Implicit attitudes can predict negative behaviors, such as discrimination and rejection, and explain why people with ASD report experiences of discrimination and social exclusion (Dickter et al., 2020).

Overall, the findings of the present study indicate that the Greek-translated and adapted SATA scale presents acceptable psychometric properties and can constitute a valid and reliable assessment tool to evaluate attitudes toward individuals with ASD. This study, which is the first validation of the questionnaire in another language than English, provides evidence about the internal consistency, reliability, and construct validity of the original scale (Flood et al., 2013). More research is needed for the subscale of Knowledge since the explanatory factor analysis loads a mixture of items from the Knowledge and the Societal Attitudes subscales, indicating that this subscale may need substantial adaptations in favor for reliably assessing knowledge about ASD. This finding is consistent with the results from the original standardization of the SATA scale (Flood et al., 2013). Furthermore, the results reveal moderate levels of knowledge and positive attitudes regarding people with ASD a consistent finding with relevant research in developing countries (Chu et al., 2021; Wang et al., 2012). Finally, the factors of gender, age, and educational level significantly affect scores on the Greek SATA scale confirming previous research (Cage et al., 2019; Chu et al., 2021; Jensen et al., 2016; Jones et al., 2021; Kuzminski et al., 2019; Wang et al., 2012).

### Limitations and Conclusions of the Study

This study had certain limitations. First, the sample was relatively small, and there was a significant gender difference between female and male participants, with women being significantly more than men (83.1% of the total sample). Thus, the results of the current study may not be entirely generalizable to the Greek population. Second, the data were collected through an online survey and included only people with access to the internet and social media. However, social media have different user demographic profiles, they are increasingly popular and play an important role in discussing societal issues among diverse audiences. Third, an important limitation is the absence of implementation of another scale assessing explicit or implicit attitudes towards disabled people or the re-administration of the Greek SATA scale after a period of time.

Despite these limitations, a key strength of the study is that the Greek version of SATA showed better psychometric properties than the original scale (Flood et al., 2013) and can be used as a reliable instrument. Finally, this study has significant value since there is scarce research investigating knowledge and attitudes towards people with ASD in Greece.

### Implications of the Study

The findings of the current study bring to the fore the need for planning interventions by policy makers to improve the knowledge and awareness about ASD in targeted groups of the Greek general population in order to prevent stigma experiences in people with ASD and their families. Basic knowledge about autism should be provided in all undergraduate programs in schools of education and health sciences. Health professionals should be sensitized to the adoption of social models for disability and abandon the medical/deficit model, while teachers should acquire a better understanding of the implementation of inclusion, as spatial integration and not inclusion is observed in Greek Schools (Pappas et al., 2018). By using television and social media, official bodies and organizations (governmental and non-governmental) can convey knowledge and reduce misinformation and stigma towards people with ASD. Furthermore, awareness campaigns and encouragement of people of all ages to participate in events or in local clubs or as volunteers in schools with children on the spectrum are necessary. The best way to gain knowledge and awareness about people with ASD is from the people themselves. Public awareness helps break down stereotypes and misunderstandings and serves as a form of emotional support for parents and caregivers. An awareness campaign, informing about the behavioral challenges that people with ASD face and focusing mostly on males, elderly, and people living in rural areas would be more beneficial.

## Appendix

**Table A1:** Communalities

Communalities	
**Items**	**Extraction**
People with autism should not engage in romantic relationships.	0.400
People with autism should have the opportunity to go to college.	0.391
People with autism should not have children.	0.374
People with autism should be institutionalized for their safety and others.	0.566
If a facility to treat people with autism opened in my community, I would consider moving out.	0.418
Individuals with autism are incapable of living on their own.	0.410
I would be afraid to be around a person with autism.	0.550
A person with autism is an emotional burden to his/her family.	0.180
I would be comfortable sitting next to a person with autism in the same class.	0.297
A person with autism is a financial burden to his/her family.	0.167
People with autism should be encouraged to marry someone with autism.	0.176
People with autism are incapable of forming relationships and expressing affection.	0.407
Children with autism should be fully integrated into mainstream classes.	0.150
I would be uncomfortable hugging a person with autism.	0.508
People with autism cannot understand other people’s feelings.	0.199
Students with autism who are mainstreamed into regular classrooms are a distraction to students without autism in that classroom.	0.387
People with autism require additional support to be successful in the workplace	0.403
People with autism tend to be violent	0.252
Mainstreaming children with autism into regular education classrooms poses a safety risk for children without autism in the same classroom	0.411
People with autism need assistance communicating with others	0.278
All individuals with autism demonstrate repetitive behaviors, such as rocking or flapping of arms or hands	0.355
I would be comfortable sharing an office with a co-worker with autism	0.653
I would be comfortable sitting next to a person with autism in a movie theatre	0.741
I would be comfortable having a person with autism living in the same building as me	0.722
I would be comfortable having a friend with autism	0.709
People with autism are capable of living normal lives (i.e., with a job, house, family, etc.)	0.420

**Table A2:** Factor loadings for the three subscales of the Attitudes towards Autism Scale

Item	Component		
	1	2	3
People with autism should be institutionalized for their safety and others.	0.725		
I would be uncomfortable hugging a person with autism.	0.676		
I would be afraid to be around a person with autism.	0.676		
If a facility to treat people with autism opened in my community. I would consider moving out.	0.638		
People with autism should have the opportunity to go to college.	0.525		
People with autism are incapable of forming relationships and expressing affection.	0.496		0.367
People with autism should not engage in romantic relationships.	0.487		0.380
Mainstreaming children with autism into regular education classrooms poses a safety risk for children without autism in the same classroom	0.466		0.378
People with autism should not have children.	0.447		0.414
People with autism should be encouraged to marry someone with autism.	0.418		
A person with autism is an emotional burden to his/her family.	0.343		
A person with autism is a financial burden to his/her family.	0.318		
I would be comfortable sitting next to a person with autism in a movie theatre		0.838	
I would be comfortable having a person with autism living in the same building as me		0.823	
I would be comfortable having a friend with autism		0.805	
I would be comfortable sharing an office with a co-worker with autism		0.772	
I would be comfortable sitting next to a person with autism in the same class.		0.501	
People with autism require additional support to be successful in the work place			0.626
All individuals with autism demonstrate repetitive behaviors. such as rocking or flapping of arms or hands			0.592
Students with autism who are mainstreamed into regular classrooms are a distraction to students without autism in that classroom.			0.557
People with autism need assistance communicating with others			0.527
Individuals with autism are incapable of living on their own.	0.374		0.515
People with autism are capable of living normal lives (i.e. with a job. house. family. etc.)		0.421	0.477
People with autism tend to be violent			0.417
Children with autism should be fully integrated into mainstream classes.			0.376
People with autism cannot understand other people’s feelings.			0.371

*The different color shadings represent items from the following subscales*:

- *Societal attitudes*

- *Knowledge*

- *Personal distance*
