# Correction: Societal Attitudes Towards Autism (SATA): Validation of the Greek Version in the General Population

**DOI:** 10.1007/s10803-026-07264-w

**Published:** 2026-02-18

**Authors:** Vasiliki Zarokanellou, Alexandros Gryparis, Paraskevi Papatheodorou, Giorgos Tatsis, Dionysios Tafiadis, Angelos Papadopoulos, Louiza Voniati, Vassiliki Siafaka

**Affiliations:** 1 Department of Speech and Language Therapy, School of Health Sciences, University of Ioannina, 4th Km National Road Ioannina-Athens, 45500 Ioannina, Greece; 2 Karamandaneio Prefecture Children Hospital of Patras, Patras, Greece; 3 Department of Speech & Language Therapy, Faculty of Sciences, European University, Nicosia, Cyprus

**Correction to: Journal of Autism and Developmental Disorders (2022) 54:1582–1593**

10.1007/s10803-022-05842-2

This erratum serves to acknowledge related prior work by Raissa Kouznetsov, whose master’s thesis, “Investigating the general population’s knowledge, attitudes, and behaviours toward people with autism (Autistic Spectrum Disorders) in Greece” [1], employed the SATA scale. In that work, the 16-item SATA scale was linguistically and culturally adapted into Greek in accordance with World Health Organization guidelines, and its psychometric properties were evaluated.
